# Combined Typhoid and Malarial Infection

**Published:** 1899-02

**Authors:** 


### COMBINED TYPHOID AND MALARIAL INFECTION.

Irving Phillips Lyon, M. D., *Am. Jour. Med. Sci.*, Jan. 1899. The paper is based on the reports of thirty cases that meet the following conditions:

1. That typhoid fever and malarial fever coexist in the same individual.

2. That this coexistence be shown (a) by the discovery of the parasites of malarial disease in the blood, and (b) by strong evidence clinical, pathological, bacteriological, etc., of true typhoid fever.

The advocates of the disease known as "typho-malarial fever" claim, as a rule, in America, that it is a prolonged fever, exhibiting typhoid characteristics, and ushered in and often accompanied during its course by repeated paroxysms of chill, high fever, and sweating. The onset of the disease is more abrupt than that of simple typhoid fever, and its termination is often by crisis. Bilious symptoms are frequent. Jaundice, or an icteroid hue of the skin, is more common. Abdominal tenderness, distensions, etc., are usually present, though often slight. Diarrhea may or may not be present. Rose-spots seldom develop. The course of the fever is shorter and markedly milder than that of typhoid fever. The prognosis is highly favorable and the mortality very low. Severe symptoms, such as hemorrhage, perforation, etc., sometimes, though rarely, occur, as in simple typhoid fever. As to the value of quinine much diversity of opinion is found. All agree that it does not influence the course of the typhoid element, and many state that its influence in controlling the malarial manifestions is slight or entirely wanting. Others state that it is of distinct value in combating the malarial element. Most of the French writers state that the course of the disease is usually severe and the mortality high; that complications are common; that rose-spots usually appear; that quinine has a modifying influence on the disease. Some French writers hold that the two diseases are merely superimposed, the one upon the other, each preserving its individuality, and others find that the clinical picture varies markedly in individual cases. As a rule the Italian observers hold that the two diseases are coincident and superimposed, each preserving its individuality. It is thus seen that the American description of "typo-malarial fever" differs essentially from the French.

A glance at the recorded cases shows that, as a rule, the course of the disease was of at least average severity as compared with typhoid fever and generally of greatly increased severity. Only one or two cases exhibited a mild course. The mortality was high. Ten of the thirty cases ended fatally, a mortality of 33.3 per cent. This high rate of mortality is nearly double that of typhoid fever, if we take a large body of typhoid statistics covering a long period of years and collected from different climates and regions of the world. Complications, often severe, are seen to have occurred in a large number of the cases. Rose-spots are specifically mentioned in twenty of the cases, and in several of the other cases they may have occurred, although the notes are silent on this point. Quinine was given on the appearance of malarial manifestations in nearly all, if not, in fact, in all of the cases, and is seen to have had an unfailing influence, either controlling the malarial element for a period or removing it entirely from the scene. Quinine thus shows itself to be a true remedial agent, and appears to be as efficacious in controlling the malarial element in the combined infection as it is in uncomplicated cases of malarial fever. It is also of diagnostic value, second only in importance to the microscopical examination of the blood.

As to the clinical picture presented, no uniformity is found. The typhoid picture, however, was always present, and in nearly all of the cases dominated the scene. The malarial manifestations appeared at different points in the different cases. Repeated paroxysms of chill, fever, and sweating at the outset of the disease appeared in many of the cases, in this respect resembling the picture generally described in "typho-malarial" fever. In other cases these features were absent. In some of the cases febrile paroxysms occurred during the height of the disease and irregularly through its course, though this was the exception. In other cases the malarial paroxysms occurred only in the convalesence from the typhoid. It is interesting to note also that in several cases malarial paroxysms directly preceded or accompanied the onset of the typhoid, disappeared completely from view during its course and again reappeared during convalescence.

In these cases, then, the observations of the French, rather than of the American observers, are sustained. The important point of difference, clinically between the "typho-malarial fever" of the French and of the Americans is found in the severity and mortality of the disease. The probability is that the cases described by Southern writers as "typho-malarial" will prove, when scientific methods are used in studying the case, to be either mild cases of simple typhoid or continued malarial fever. The cases where there is a true combining of the two diseases is very rare, in Johns Hopkins Hospital where hundreds of cases of each disease are treated only two such cases having been observed. In tropical countries, where malaria is more common and typhoid prevails, the coincidence may occur more commonly.—*North Carolina Medical Journal.*

